# Canine Coronavirus Activates Aryl Hydrocarbon Receptor during In Vitro Infection

**DOI:** 10.3390/v14112437

**Published:** 2022-11-03

**Authors:** Claudia Cerracchio, Francesco Serra, Maria Grazia Amoroso, Filomena Fiorito

**Affiliations:** 1 Department of Veterinary Medicine and Animal Production, University of Naples Federico II, 80137 Naples, Italy; 2 Department of Animal Health, Unit of Virology, Istituto Zooprofilattico del Mezzogiorno, 80055 Portici, Naples, Italy

**Keywords:** CCoV, A72 cells, AhR, CH223191, NP, virus yield

## Abstract

The aryl hydrocarbon receptor (AhR) is a ligand-activated transcription factor that interacts with substrates, including microbial metabolites. Recent advances reveal that AhR is involved in the host response to coronaviruses (CoVs) infection. Particularly, AhR antagonists decrease the expression of angiotensin-converting enzyme 2 (ACE2) via AhR up-regulation, resulting in suppression of severe acute respiratory syndrome coronavirus-2 (SARS-CoV-2) infection in mammalian cells. Herein, we report that AhR is expressed in canine fibrosarcoma (A72) cells, where it is considerably activated by infection with genotype II of canine coronavirus (CCoV-II). The pharmacological inhibition of AhR, by CH223191, suppressed cell death signs and increased cell viability. Furthermore, the AhR antagonist induced a meaningful decline in virus yield, accompanied by the inhibition of the expression of viral nuclear protein (NP). Fascinatingly, during CCoV infection, a novel co-expression of NP and AhR expression was found. Taken together, our preliminary findings show that infection with CCoV activates AhR, and pharmacologic AhR inhibition reduces CCoV replication, identifying AhR as a possible candidate target for CCoV antiviral therapy.

## 1. Introduction

Coronaviruses (CoVs) are a large group of positive-stranded RNA viruses which can infect several hosts such as mammals and birds. They are able to mutate into new and more dangerous strains. Indeed, the propensity of CoV genomes to mutate and recombine allows them to overcome natural barriers preventing cross-species transmission, to adapt and proliferate in new species in a spillover phenomenon [1,2,3,4]. Therefore, novel strains emerge through molecular processes that affect their adaptation, transmissibility, host/tissue tropism, and pathogenicity. Genotype II of canine coronavirus (CCoV-II), an alphacoronavirus, belongs to the subfamily of *Orthocoronavirinae*, family of *Coronaviridae*, order of *Nidovirales*. CCoV-II is generally responsible for self-limiting enteric infections, causing high morbidity and low mortality in dogs [1,2,3,4]. However, an extremely virulent CCoV-IIa strain (CB/05) has been detected from Italian outbreaks of fatal disease in puppies, due to multi-systemic infections with severe lesions in different organs [5]. Genetic analysis highlighted a recombinant canine–feline–porcine origin of CB/05 due to a partial S-gene exchange with transmissible gastroenteritis virus of swine (TGEV) [3,4,6,7,8]. Recently, a new CCoV has been isolated in Malaysia from a child hospitalized for pneumonia between 2017 and 2018 [9]. Genome sequencing has been identified it as a novel canine–feline recombinant alphacoronavirus (genotype II), called CCoV-HuPn-2018 [9]. In addition, a novel CoV, named HuCCoV_Z19Haiti, has been isolated from the urine samples of a medical team member presenting mild fever and malaise after a travel to Haiti. The virus showed 99.4% similarity with the recombinant CCoV-HuPn-2018 identified in Malaysia [10]. Interestingly, as reviewed in the study by Vlasova et al., 2022 [11], recombinant canine–feline–porcine alphacoronaviruses have been also found in humans in Thailand [12], as well as in USA [13], proposing that these viruses emerged in multiple geographic locations independently [11].

AhR is a transcription factor activated by endogenous and exogenous substrates, including bilirubin, biliverdin, tryptophan metabolites, environmental pollutants (such as dioxin), and microbial metabolites [14,15]. It is differently expressed in almost all mammalian cells [15]. In the canonical pathway of AhR action, after ligand binding, the complex AhR-ligand is transferred inside the nucleus, in which the nuclear translocator the heterodimer of ARNT and AhR is responsible for the interaction with specific sequences of DNA, for controlling the expression of target genes, such as those encoding the cytochrome P450 enzymes (e.g., CYP1A1, CYP1B1, and CYP2A1). As result, the release of cytokines and the control of immune response occur [15]. During infection, AhR regulates some aspects of the immunity, by interfering with natural protective immune responses to different microorganisms [16,17,18]. Indeed, AhR inhibits the production of type I interferons (IFN-I) [19], specifically, during infection with Zika or dengue viruses [20]. In addition, it has been shown the involvement of AhR in the antiviral property of 3-O-methylfunicone, a secondary metabolite produced by fungus *Talaromyces pinophilus*, during bovine herpesvirus 1 infection [21]. AhR is also involved in the host response to different CoVs. AhR activation has been revealed during infection with murine coronavirus (MCoV), Middle East respiratory syndrome coronavirus (MERS-CoV), severe acute respiratory syndrome (SARS-CoV-1), SARS-CoV-2, and human coronavirus (HCoV) 229E [22,23,24]. Specifically, during coronavirus-induced disease 2019 (COVID-19) SARS-CoV-2-induced, AhR modulates the expression of ACE-2 as well as its stabilizing partner, the broad neutral amino acid transporter 1 (B0AT1) [24,25], suggesting that AhR up-regulation is involved in promoting viral replication [24]. Furthermore, an AhR antagonist (CH223191) inhibits the replication in vitro of SARS-CoV-2 and HCoV-229E [24]. Based on these findings, herein, we report that AhR, expressed in canine A72 cells, is activated by infection with CCoV-II. Additionally, pharmacologic AhR inhibition repressed CCoV replication, recognizing AhR as a likely target for CCoV antiviral therapy. We used A72, a canine fibrosarcoma cell line, which is suitable for studying CCoV, since the primary canine kidney cells show different susceptibility to this virus [26].

## 2. Materials and Methods

### 2.1. Cell Cultures and Virus Infection

A72, a canine fibrosarcoma cell line, was maintained in Dulbecco’s modified Eagle’s minimal essential medium (DMEM) and incubated at 37 °C and 5% CO_2_ [27,28]. CCoV type II, strain S/378, kindly provided by Prof. C. Buonavoglia (University of Bari Aldo Moro, Italy) was utilized throughout the study. A72 cells were used both for virus stocks growth and for virus titration [27].

2-methyl-2H-pyrazole-3-carboxylic acid (2-methyl-4-o-tolylazo-phenyl)-amide (CH223191) (Sigma-Aldrich, St. Louis, MI, USA), a synthetic and specific AhR competitive antagonist [15,29], was solubilized in DMSO (Sigma-Aldrich, St. Louis, MI, USA) and used at a concentration of 2, 5, 10 and 20 μM.

Monolayers of A72 cells were pretreated for 1 h at 37 °C with DMEM 10% FBS containing different concentrations of CH223191 (2, 5, 10 and 20 μM). Then, cells were infected or not with CCoV, at a multiplicity of infection (MOI) of 0.05 or 5. After 1 h of adsorption at 37 °C, cells were incubated and processed at 24 h post infection (p.i.). CCoV was in culture medium throughout the course of the experiment.

### 2.2. Cell Viability

Cell viability was evaluated by trypan blue (TB) (Sigma-Aldrich) exclusion test [21,30]. In brief, monolayers of A72 cells, pretreated or not with CH223191 at various doses (2, 5, 10 and 20 μM), were infected or not with CCoV at MOI of 0.05, and after 24 h of treatment, cell viability was assessed by trypan blue while cells were attached to wells, as previously described [31,32]. Cell viability was determined as percentage of living cells over total cell number. Results are shown as the mean ± S.D. of three independent experiments performed in duplicate.

### 2.3. Examination of Cell Morphology

To study cell morphology, light microscopy after Giemsa staining was utilized [21,32]. Monolayers of A72, pretreated or not with CH223191, were infected or not with CCoV, at an MOI of 0.05 and 5, and incubated at 37 °C. After 24 h of infection, Giemsa staining was assessed, and light microscopy analysis was performed under ZOE Cell Imager (Bio-Rad Laboratories, Segrate, Milan, Italy). Cell death features were detected by using the criteria previously described [33,34,35,36].

### 2.4. Immunofluorescence Staining

Monolayers of A72, pretreated or not with CH223191, were infected or not with CCoV, at an MOI of 0.05 and 5, and incubated at 37 °C. After 24 h p.i., immunofluorescence staining was evaluated as previously reported [21,37], by using the following antibodies, diluted in 5% bovine serum albumin-1X Tris-Buffered Saline, 0.1% Tween^®^ 20 Detergent: anti-AhR (Sigma-Aldrich, St. Louis, MI, USA) (1:250), anti-NP monoclonal mouse, MAB 938 (The Native Antigen Company, Kidlington, UK), Alexa Fluor 488 goat anti-mouse (Thermo Fisher Scientific, Waltham, MA, USA) (1:1000), and Texas Red goat anti-rabbit (Thermo Fisher Scientific, Waltham, MA, USA) (1:100). Microscopy and photography were assessed by ZOE Fluorescent Cell Imager (Bio-Rad Laboratories, Hercules, CA, USA). Quantification of fluorescence signals from microscopy-generated images was performed using ImageJ (National Institutes of Health, Bethesda, MD, USA) software.

### 2.5. Virus Infection

Monolayers of A72, pretreated or not with CH223191, were infected or not with CCoV, at an MOI of 0.05 and 5, incubated at 37 °C, and processed after 24 h of infection by real-time PCR for CCoV quantification. Furthermore, viral cytopathic effect (CPE) was evaluated, by examining cells under a light microscope at 24 h p.i. [21,27].

### 2.6. Viral Nucleic Acids Extraction Procedures

Nucleic acid extraction was carried out from 200 µL of cell supernatant by using the King Fisher Flex System (Thermo Fisher Scientific, Waltham, MA, USA) with the Mag Max Viral Pathogen kit (Thermo Fisher Scientific, Waltham, MA, USA), according to the manufacturer’s instructions. Nucleic acids were eluted in 60 µL of elution buffer. DMEM was utilized as a negative process control.

### 2.7. CCoV Viral Load Quantification by Real-Time Reverse Transcription PCR (RT-qPCR)

CCoV was quantified in all the samples by RT-qPCR. Detection was carried out on a QuantStudio 5 Real-Time PCR thermal cycler (Thermo Fisher Scientific, Waltham, MA, USA) in a total volume of 25 µL containing 5 µL of nucleic acid extract, 12.5 µL of AGPATH reaction kit with 1 µL of reverse transcriptase enzyme (Thermo Fisher Scientific, Waltham, MA, USA), 1 µL (10 µM) of primer forward CCoV-For (5′-TTGATCGTTTTTATAACGGTTCTACAA-3′), 1 µL (10 µM) of primer reverse CCoV-Rev (5′-AATGGGCCATAATAGCCACATAAT-3′) and 1 µL (6 µM) of probe CCoV-P (FAM-5′-ACCTCAATTTAGCTGGTTCGTGTATGGCATT-3′-TAMRA). The thermal profile was the following: reverse transcription for 30 min. at 42 °C, initial denaturation for 15 min at 95 °C, 40 cycles of amplification for 15 s at 95 °C and for 60 s at 60 °C [38]. Quantification was carried out by a standard curve, developed by amplifying serial dilutions of the quantified extracted virus (from 3.5 × 10^9^ to 3.5 × 10^4^ TCID_50_/mL) and plotting the Log TCID_50_/mL versus the C_t_ number [21].

### 2.8. Statistical Analysis

Data are described as mean ± S.D. One-way ANOVA with Tukey’s post-test and by Student’s t test was calculated by GraphPad InStat Version 3.00 for Windows 95 (GraphPad Software, San Diego, CA, USA). *p* < 0.05 was considered statistically significant.

## 3. Results

### 3.1. AhR Inhibitor Increases Cell Viability during CCoV Infection

**Figure 1:**
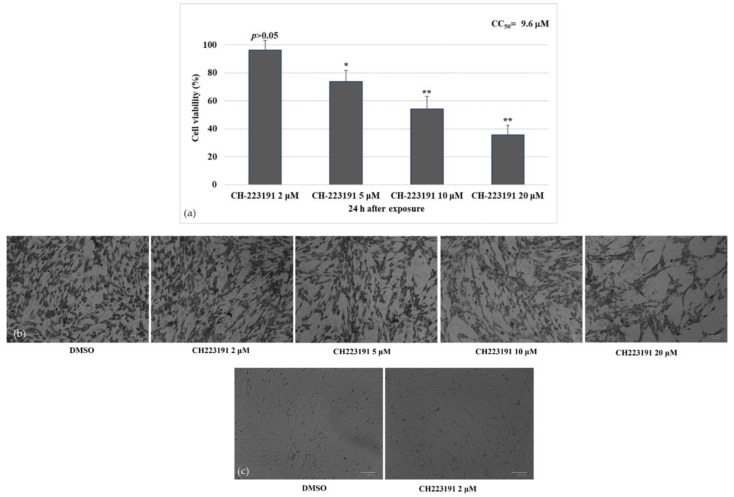
Identifying CC_50_ of CH223191 inhibitor at different doses and developing dose–response curve in A72 cells. (**a**) Dose–response curve of A72 cells treated with CH223191 at different concentrations (2, 5, 10 and 20 μM). After 24 h of treatment, cell viability was determined by TB staining while cells were attached to wells and counted under a light microscope. Significant differences between DMSO and CH223191-treated cells are indicated by probability *p*. * *p* < 0.05 and ** *p* < 0.01. (**b**) At 24 h after treatment, cells were stained with Giemsa and observed under a light microscope. (**c**) A72 cells treated with DMSO or with CH223191 (2 μM). Scale bar 100 µm.

To investigate the effect of CH223191 during CCoV infection in A72 cells, cell viability was evaluated by Trypan Blue exclusion test. Thus, we first tested the effects of four different concentrations of CH223191 (2, 5, 10 and 20 μM) on A72 uninfected cells. After 24 h of treatment, we identified the cytotoxic concentration value required to reduce cell viability by 50% (CC_50_) of CH223191 and developed dose–response curve (Figure 1a). Cell viability (% control) was detected in A72 cells with a CC_50_ of 9.6 μM CH223191, after 24 h of treatment (Figure 1a). CH223191 at 2 µM in A72 cells produced no significant differences in cell viability (*p* > 0.5) (Figure 1a–c).

**Figure 2:**
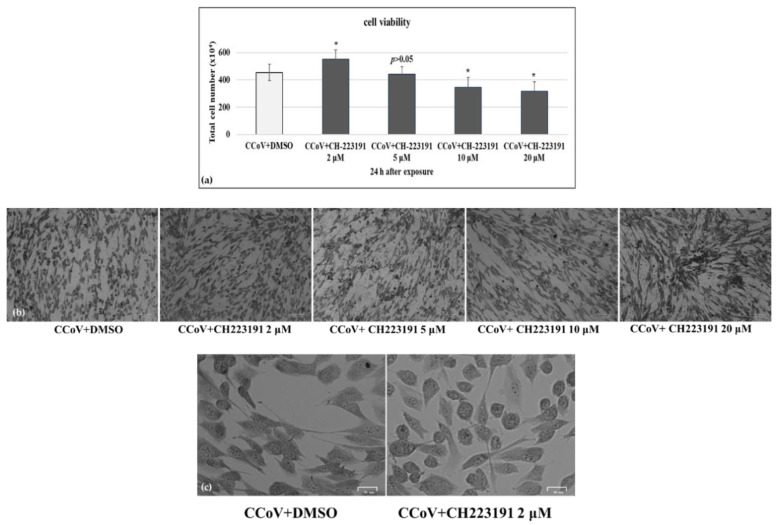
AhR inhibitor CH223191 increases cell viability during CCoV infection. (**a**) Dose–response curve of A72 cells treated with CH223191 at different concentrations (2, 5, 10 and 20 μM). After 24 h of treatment, cell viability was determined by TB staining while cells were attached to wells and counted under a light microscope. Significant differences between CCoV+DMSO and CCoV+CH223191-treated cells are indicated by probability *p*. * *p* < 0.05. (**b**) A72 cells infected with CCoV and treated or untreated with CH223191 at different concentrations (2, 5, 10 and 20 µM). At 24 h after treatment, cells were stained with Giemsa and observed under a light microscope. Scale bar 100 µm. (**c**) A72 cells infected with CCoV and treated or untreated with CH223191 (2 µM). At 24 h after treatment, cells were stained with Giemsa and observed under a light microscope. Scale bar 25 µm.

Then, monolayers of A72 cells were infected with CCoV at MOI of 0.05 and were exposed or not to CH223191 at different doses (2, 5, 10 and 20 μM). Following CCoV infection in A72 cells, we detected a significant (*p* < 0.05) increase in cell viability in the presence of CH223191 at 2 µM (Figure 2a–c).

Thus, the concentration of CH223191 at 2 µM was selected to be used throughout the study. Our findings showed that in A72 cells, at the non-toxic concentration of 2 µM, CH223191 significantly decreased cell death after 24 h of CCoV infection.

### 3.2. AhR Inhibitor Reduces Morphological Cell Death Features during CCoV Infection in A72 Cells

**Figure 3:**
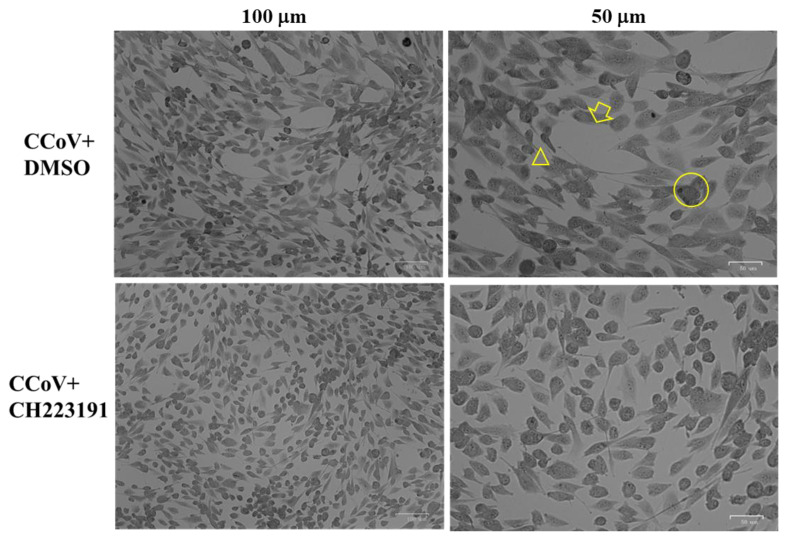
AhR inhibitor CH223191 reduces morphological cell death signs during CCoV infection in A72 cells. Cells were infected with CCoV, in the presence or absence of CH223191. At 24 h p.i., cells stained by Giemsa were analyzed under a light microscope. Photomicrographs showing in unexposed infected groups, some cells with cell death features, such as an increase in intercellular spaces due to detachment from culture plate (arrow). In addition, morphological apoptotic marks, such as cell shrinkage (arrowhead), pyknosis and chromatin condensation (circle), were detected. In the presence of AhR inhibitor, all those cell death features were markedly diminished in CCoV-infected cells.

To explore the effects of AhR inhibitor CH223191 during CCoV infection, morphological examination of A72 cell was performed through light microscopy after Giemsa staining at 24 h of infection. As displayed in Figure 1b, the lowest concentration of AhR inhibitor (2 μM) did not cause morphological changes on uninfected cell groups compared to control, whereas in unexposed infected cells, we found an increase in intercellular spaces due to detachment from culture plate (Figure 3, arrow). These features were accompanied by a change in cell morphology suggesting signs of apoptosis, such as cell shrinkage (Figure 3, arrowhead), pyknosis and chromatin condensation (Figure 3, circle). All those cell death marks were markedly (19%) diminished when CCoV-infected cells were treated with AhR inhibitor (Figure 3).

Overall, these findings demonstrated that CH223191 remarkably protected A72 cells during CCoV infection.

### 3.3. A72 Cells Express AhR

**Figure 4:**
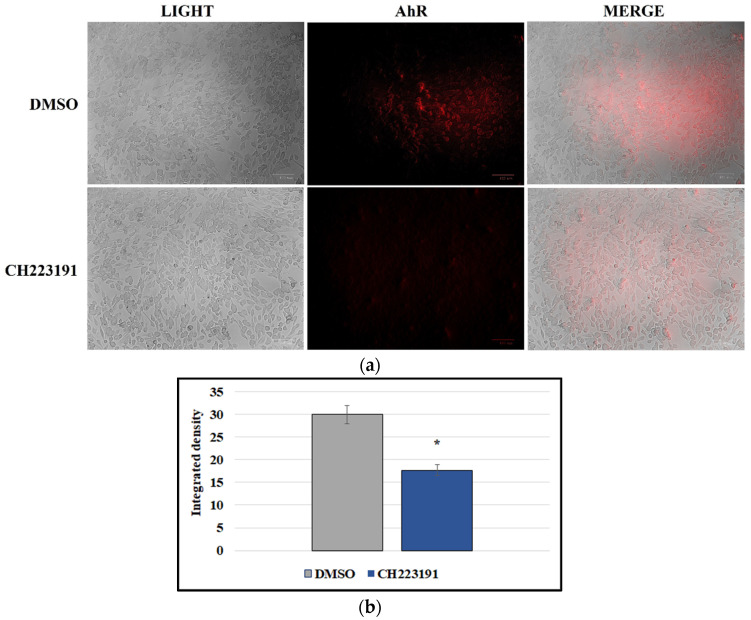
A72 cells express AhR. (**a**) Canine fibrosarcoma cell line A72 expressed AhR. AhR inhibitor CH223191 noticeably decreased the expression of AhR. Scale bar 100 µm. (**b**) Bars represent the mean ratio generated from the integrated density (product of the area and mean intensity of fluorescence) of the AhR expression evaluated by ImageJ. Error bars represent standard deviation measurement. Significant differences between CCoV-infected cells and AhR-inhibitor-treated infected cells are indicated by probability *p*. * *p* < 0.05.

In order to investigate the expression of AhR in A72 cells, immunofluorescence staining was performed. As displayed in Figure 4a, AhR was expressed by the canine fibrosarcoma cell line A72. Interestingly, AhR inhibitor CH223191 considerably decreased the expression of AhR (Figure 4a), as confirmed by the measurement of integrated density fluorescence (Figure 4b).

### 3.4. CCoV Infection Activates the Expression of AhR in A72 Cells

**Figure 5:**
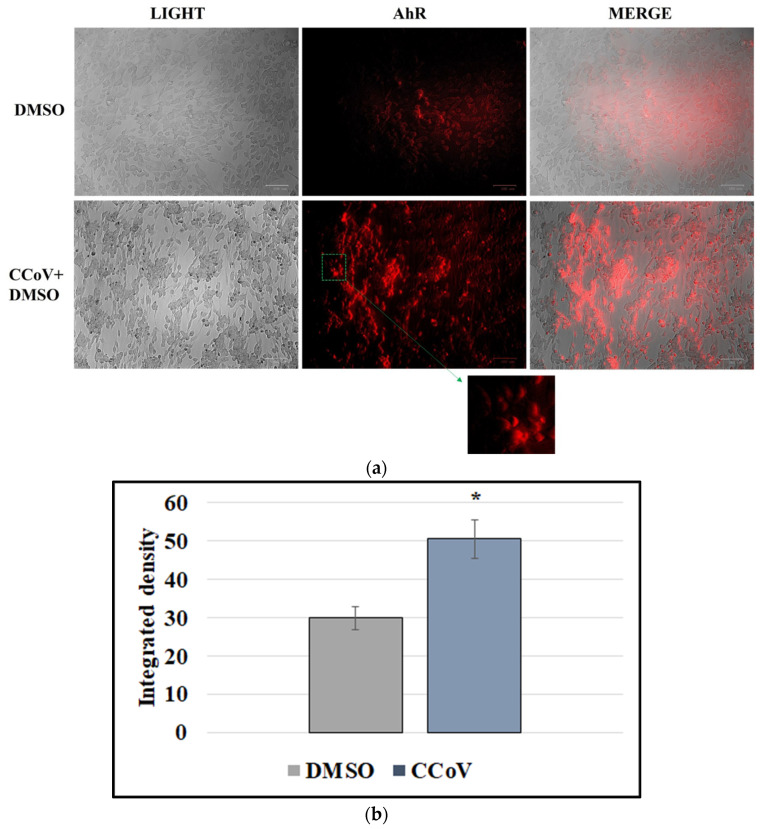
CCoV infection activates the expression of AhR in A72 cells. A72 cells were infected with CCoV at MOI of 0.05. At 24 h p.i., immunofluorescence staining for AhR was performed. (**a**) In CCoV-infected cells a significant increase in AhR expression was found. Scale bar 100 µm. (**b**) Bars represent the mean ratio generated from the integrated density (product of the area and mean intensity of fluorescence) of the AhR expression during CCoV infection evaluated by ImageJ. Error bars represent standard deviation measurement. Significant differences between CCoV-infected cells and AhR-inhibitor-treated infected cells are indicated by probability *p*. * *p* < 0.05.

To examine the expression of AhR during CCoV infection, we performed immunofluorescence staining. In CCoV-infected cells, we detected a significant increase in AhR expression (Figure 5a). This finding was confirmed by integrated density measurement, indicating that up-regulation of AhR by CCoV was 1.7 times higher compared to uninfected cell group (Figure 5b).

### 3.5. AhR Inhibitor Inhibits Both AhR and NP Expression during CCoV Infection in A72 Cells

**Figure 6:**
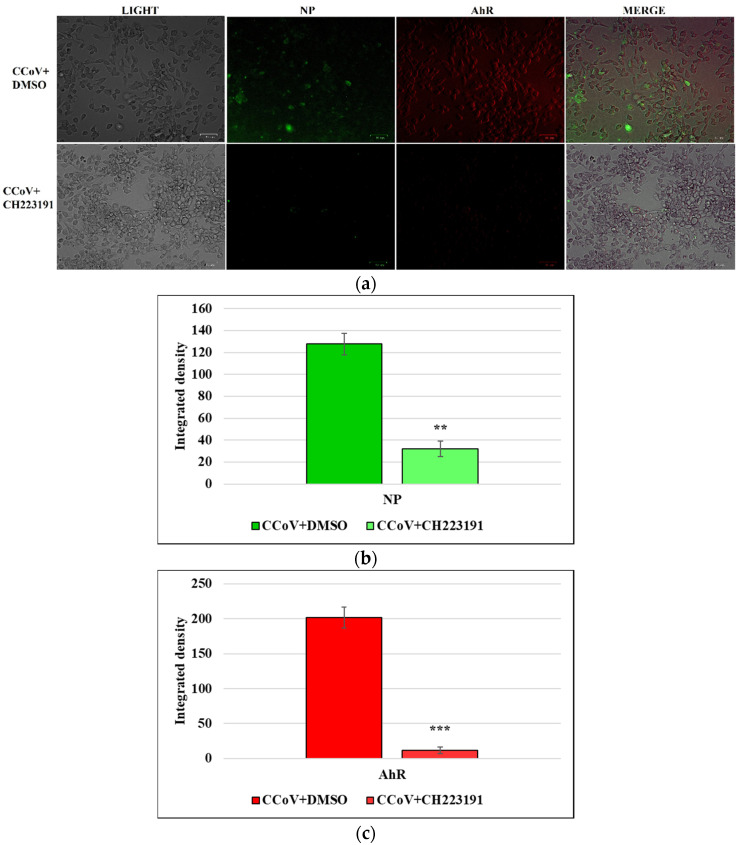
AhR inhibitor inhibits both AhR and NP expression during CCoV infection in A72 cells. A72 cells were infected with CCoV at MOI of 5. At 24 h p.i., immunofluorescence staining for AhR and NP was performed. (**a**) In CCoV-infected cells a significant up-regulation of AhR and NP expression was detected. In some merged images of A72 cells, co-expression of NP and AhR expression was found (MERGE). Following infection, in the presence of AhR inhibitor CH223191, both AhR and NP expression was down-regulated. Scale bar 59 µm. (**b**) Bars represent the mean ratio generated from the integrated density (product of the area and mean intensity of fluorescence) of the NP expression during CCoV infection evaluated by ImageJ. Error bars represent standard deviation measurement. Significant differences between CCoV-infected cells and AhR-inhibitor-treated infected cells are indicated by probability p. ** *p* < 0.01. (**c**) Bars represent the mean ratio generated from the integrated density (product of the area and mean intensity of fluorescence) of the AhR expression during CCoV infection evaluated by ImageJ. Error bars represent standard deviation measurement. Significant differences between CCoV-infected cells and AhR-inhibitor-treated infected cells are indicated by probability *p*. *** *p* < 0.001.

Simultaneously, the expression of AhR and NP during CCoV infection on A72 cells were tested. NP was expressed during CCoV infection, and we also found that there was a co-expression of NP and AhR in some merged images of A72 cells (Figure 6a-MERGE). Moreover, using AhR inhibitor CH223191 not only down-regulated the expression of AhR, but also of NP (Figure 6a). Those findings were confirmed by integrated density fluorescence measurement (Figure 6b,c).

Overall, these findings suggest that not only CCoV infection activated AhR expression, but in some cells, co-expression of NP and AhR was detected. Interestingly, using AhR inhibitor, the down-regulation of both NP and AhR was found.

### 3.6. AhR Inhibitor Decreases Virus Yield during CCoV Infection in A72 Cells

#### 3.6.1. CPE Evaluation

Following CCoV infection at MOI 0.05 in A72 cells, at 24 h p.i. an increase in CPE was found (Figure 2b,c and Figure 3). Indeed, morphological alterations, such as development in syncytia of giant cells and detachment from culture plate in CCoV-infected groups, were detected (Figure 2b,c and Figure 3). These features were intensely reduced by the presence of AhR inhibitor (Figure 2b,c and Figure 3).

#### 3.6.2. Standard Curve and Virus Quantification

**Figure 7:**
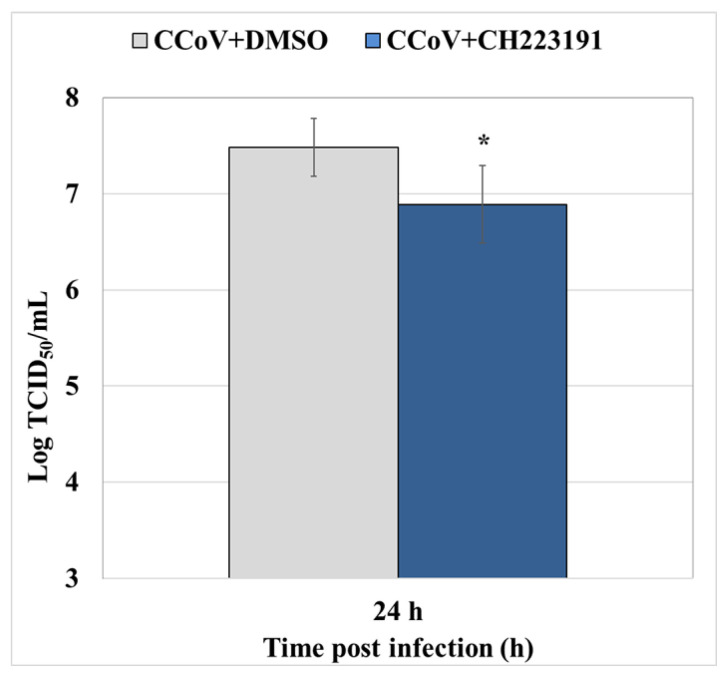
AhR inhibitor decreases virus yield during CCoV infection in A72 cells. Cells were infected with CCoV in the presence or absence of AhR inhibitor CH223191. At 24 h p.i., virus yield was evaluated by RT-qPCR by the mean of a standard curve created plotting Log TCID_50_/mL against the C_t_ number. Significant differences between CCoV-infected cells and AhR-inhibitor-treated infected cells are indicated by probability *p*. * *p* < 0.05.

AhR ability to decrease virus yield was evaluated also by Rt-qPCR. Quantification was made by the mean of a standard curve. The standard curve was constructed based on the average C_t_ values of three replicates against the Log of known amount of the virus (expressed in TCID_50_/mL). The resulting equation was y= −3.379x + 47.542 (E = 97%, r^2^ = 0.998). The standard curve was used in RT-qPCR assays [21] to estimate samples’ virus titer starting from the C_t_ obtained in the amplification reaction. Our results showed that CCoV virus yield was significantly reduced by AhR inhibitor in A72 cells (Figure 7).

Taken together, our results demonstrated that AhR inhibitor significantly reduced CCoV virus yield during infection in A72 cells.

## 4. Discussion

The ability of CoVs to mutate and recombine their genome has been extensively proved. This feature allows them to cross interspecies barriers. Specifically, recent studies highlight the aptitude of CCoV, an alphacoronavirus, to mutate into new and more dangerous variants [1,3,4,11]. In different countries of the world, and not simultaneously, extremely virulent recombinant canine–feline–porcine alphacoronaviruses have been identified in dogs as well as in humans [1,3,4,11]. These findings indicate the need for constant surveillance of CoVs, such as CCoV. 

To date, indomethacin, an anti-inflammatory drug, has been demonstrated to have effective antiviral properties against CCoV infection, acting on the virus replication cycle and blocking viral RNA synthesis [39,40,41]. Recent advances reveal the involvement of AhR during CoVs infection [22,23,24]. In particular, AhR antagonist, like CH223191, decreases the expression of the angiotensin-converting enzyme 2 (ACE2) receptor, via AhR activation, resulting in the suppression of SARS-CoV-2 infection in mammalian cells [24]. Herein, first, we found that AhR was expressed in canine A72 cells. Moreover, it was significantly activated during CCoV infection, as reported in other CoVs infections [22,23,24]. In fact, the measurement of integrated density fluorescence showed that the expression was 1.7 times higher in infected cell groups compared to uninfected cells. 

Using a well-known AhR inhibitor, CH223191, at dose of 2 μM did not cause toxic effects in A72 cells, and significantly reduced cell death after 24 h of CCoV infection. In addition, CH223191 markedly diminished morphological cell death features, typical marks of CCoV infection in A72 cells [27,42]. Those findings were accompanied by a significant decline in virus yield, a result supposing that AhR up-regulation may represent a common approach used by CoVs to stimulate viral replication [22,23,24] (see Table 1).

Following CCoV infection, in the presence or in the absence of AhR inhibitor, the expression of both NP and AhR were simultaneously tested. Surprisingly, in some A72 infected cells, a novel co-expression of NP and AhR expression was found, and the possible explanations for that finding need further investigation. Furthermore, the AhR inhibitor not only significantly down-regulated the expression of AhR, but also of NP. Evidence on structural knowledge as well as on the functional mechanism of viral nucleocapsid protein NP is considered a starting point for the development of potential inhibitors against CoV diseases. Indeed, NP is responsible of binding the viral RNA genome, packing viral genome RNA into ribonucleoproteins, and compressing it into a compact virion core [43]. Moreover, it is generally more stable than the CoV spike protein, which has a higher mutation rate [44,45,46].

Overall, based on our results, emphasizing the possible role of AhR as a target for antiviral therapy, we suppose that some AhR ligands may improve the host response to CoVs infection. Additionally, we identified NP antagonists for targeting not only CCoV but also human CoVs. Future biochemical and structural studies may better delineate the current research. However, these results highlight the importance of screening hypothetical antivirals in an in vitro animal model of CoVs to avoid the manipulation of extremely dangerous human CoVs (SARS-CoVs and MERS-CoV).

## 5. Conclusions

Our preliminary findings indicate that AhR up-regulation might be a common stratagem used by coronaviruses to stimulate viral replication. Pharmacologic inhibition of AhR by CH223191 repressed CCoV replication, recognizing AhR as a new target for identifying antiviral drugs to counteract CoVs. Importantly, animal coronaviruses, such as CCoV, represent a valid alternative for carrying out preliminary studies on the efficacy of drugs, bypassing the risks of using a highly pathogenic and contagious virus for the first step of screening.

## Figures and Tables

**Table 1:** Summary of studies describing AhR up-regulation following infection with some Alphacoronaviruses and Betacoronaviruses.

Host	Genus	CoVs	Model	Cell Type	References
Mouse	Betacoronavirus	M-CoV	In vitro	Bone-marrow-derived macrophage	[23]
Mouse	Betacoronavirus	M-CoV	In vitro	Bone-marrow-derived dendritic cells	[23]
Mouse	Betacoronavirus	M-CoV	In vitro	Liver (C57BL-6 mice)	[23]
Mouse	Betacoronavirus	M-CoV	In vitro	Bone-marrow-derived macrophage	[24]
Human	Alphacoronavirus	H-CoV-229E	In vitro	Human hepatoma (Huh7)	[22]
Human	Alphacoronavirus	H-CoV-229E	In vitro	Human lung adenocarcinoma (A549)	[24]
Human	Betacoronavirus	MERS-CoV	In vitro	Human lung adenocarcinoma (Calu-3)	[24]
Human	Betacoronavirus	SARS CoV-1	In vitro	Human hepatoma (Huh7)	[22]
Human	Betacoronavirus	SARS-CoV-2	In vitro	Primary human lung epithelium (NHBE)	[24]
Human	Betacoronavirus	SARS-CoV-2	In vitro	Human lung adenocarcinoma (A549)	[24]
Human	Betacoronavirus	SARS-CoV-2	In vitro	Human lung adenocarcinoma (Calu-3)	[24]
Human	Betacoronavirus	SARS-CoV-2	*Patients*	Nasal swabs	[24]
Dog	Alphacoronavirus	CCoV-IIa	In vitro	Canine Fibrosarcoma (A72)	This study

## Data Availability

Not applicable.
